# TRIM25 and DEAD-Box RNA Helicase DDX3X Cooperate to Regulate RIG-I-Mediated Antiviral Immunity

**DOI:** 10.3390/ijms22169094

**Published:** 2021-08-23

**Authors:** Sarah C. Atkinson, Steven M. Heaton, Michelle D. Audsley, Oded Kleifeld, Natalie A. Borg

**Affiliations:** 1 Immunity and Immune Evasion Laboratory, Chronic Infectious and Inflammatory Diseases Research, School of Health and Biomedical Sciences, RMIT University, Bundoora, VIC 3083, Australia; sarah.atkinson@monash.edu (S.C.A.); michelle.audsley@monash.edu (M.D.A.); 2 Infection & Immunity Program, Monash Biomedicine Discovery Institute and Department of Biochemistry and Molecular Biology, Monash University, Clayton, VIC 3800, Australia; steven.heaton@riken.jp; 3 Faculty of Biology, Technion-Israel Institute of Technology, Haifa 32000, Israel; okleifeld@technion.ac.il

**Keywords:** ubiquitination, TRIM25, E3 ligase, DDX3X, DEAD-box helicase, antiviral immunity, RLR signalling, IFN, influenza, NS1

## Abstract

The cytoplasmic retinoic acid-inducible gene-I (RIG-I)-like receptors (RLRs) initiate interferon (IFN) production and antiviral gene expression in response to RNA virus infection. Consequently, RLR signalling is tightly regulated by both host and viral factors. Tripartite motif protein 25 (TRIM25) is an E3 ligase that ubiquitinates multiple substrates within the RLR signalling cascade, playing both ubiquitination-dependent and -independent roles in RIG-I-mediated IFN induction. However, additional regulatory roles are emerging. Here, we show a novel interaction between TRIM25 and another protein in the RLR pathway that is essential for type I IFN induction, DEAD-box helicase 3X (DDX3X). In vitro assays and knockdown studies reveal that TRIM25 ubiquitinates DDX3X at lysine 55 (K55) and that TRIM25 and DDX3X cooperatively enhance *IFNB1* induction following RIG-I activation, but the latter is independent of TRIM25’s catalytic activity. Furthermore, we found that the influenza A virus non-structural protein 1 (NS1) disrupts the TRIM25:DDX3X interaction, abrogating both TRIM25-mediated ubiquitination of DDX3X and cooperative activation of the *IFNB1* promoter. Thus, our results reveal a new interplay between two RLR-host proteins that cooperatively enhance IFN-β production. We also uncover a new and further mechanism by which influenza A virus NS1 suppresses host antiviral defence.

## 1. Introduction

The retinoic acid-inducible gene-I (RIG-I)-like receptors (RLRs), which include RIG-I (also termed DDX58), sense virally-derived nucleic acids in the cytosol to initiate an antiviral immune response [1,2]. Once activated, RIG-I binds MAVS (mitochondrial antiviral signalling protein; also termed IPS-1), an adaptor protein which aggregates along the mitochondrial outer membrane and converges downstream elements of the RLR signalling apparatus, including tumour necrosis factor receptor-associated factor-3 (TRAF3), inhibitor of NF-κB kinase subunit ε (IKKε), TANK-binding kinase 1 (TBK1), and DDX3X (DEAD-box helicase 3, X-linked) [3,4,5,6]. These signals converge at interferon regulatory factors (IRFs; IRF3/7), which drive the expression of type I interferon (IFN-I; namely IFN-α and IFN-β) and type III interferon (IFN-III; namely IFN-λ) and in turn modulate the expression of hundreds of antiviral genes in infected cells, neighbouring tissues and systemically.

Regulating these protein-signalling pathways is an expanding array of accessory proteins and post-translational modifications, including ubiquitination, which is mediated by E3 ligases. Specific configurations of ubiquitin attachments determine the function, abundance or subcellular distribution of nearly all key proteins in the RLR cascade, enabling controlled upregulation or downregulation of the innate antiviral immune response [7,8,9,10]. For example, the RING (really interesting new gene)-containing E3 ligase tripartite motif 25 (TRIM25) attaches K63-linked polyubiquitin chains to the RNA-bound RIG-I receptor, which some studies show contributes to its activation (e.g., [11,12,13]). TRIM25 also attaches K48-linked polyubiquitin chains to MAVS [11,14,15,16], targeting it for proteasomal degradation and subsequently hampering IRF3 activation and type I IFN production. Several viruses, including influenza virus (IAV), respiratory syncytial virus (RSV) and severe acute respiratory syndrome-coronavirus (SARS-CoV) target TRIM25-mediated RIG-I ubiquitination to suppress IFN-I production [17,18,19]. In addition to its RING-dependent functions, TRIM25 also performs RING-independent antiviral functions that are also independent of RIG-I [20,21,22].

Here, we report a previously unrecognised interaction between two proteins belonging to the RLR pathway, TRIM25 and DDX3X. We show that TRIM25 ubiquitinates DDX3X at lysine (K) 55 and that the two proteins cooperatively induce the *IFNB1* promoter upon RLR stimulation. However, the cooperative activation of *IFNB1* gene induction is independent of TRIM25’s catalytic RING domain, and is instead dependent upon the physical interaction of TRIM25 and DDX3X. Moreover, influenza A (IAV) non-structural protein 1 (NS1) disrupts the TRIM25:DDX3X interaction, abrogating both the TRIM25-mediated ubiquitination of DDX3X and TRIM25:DDX3X cooperatively enhanced *IFNB1* promoter induction. Thus, our results reveal that TRIM25 and DDX3X have further extended roles in IFN-β expression following RIG-I activation, and a new and additional mechanism by which influenza A virus utilises its NS1 protein to suppress immune defence.

## 2. Results

### 2.1. DDX3X Is a Novel TRIM25 Binding Partner

TRIM25 is an E3 ligase that ubiquitinates multiple proteins within the RLR signalling cascade [11,16]. We performed TRIM25 co-immunoprecipitation and mass spectrometry of TRIM25 interacting partners to further understand the role of TRIM25 in cell signalling. We transfected HEK293T cells to express FLAG-TRIM25 followed by co-immunoprecipitation of TRIM25-binding partners using anti-FLAG sepharose beads. The resulting proteins were digested with trypsin and subjected to liquid chromatography–tandem mass spectrometry (LC–MS/MS) analysis. In addition to TRIM25 peptides, we identified a substantial enrichment of peptides from another protein with roles in the RLR-signalling cascade, DDX3X, and significantly these peptides covered ~17% of the DDX3X sequence (Supplementary Table S1).

**Figure 1:**
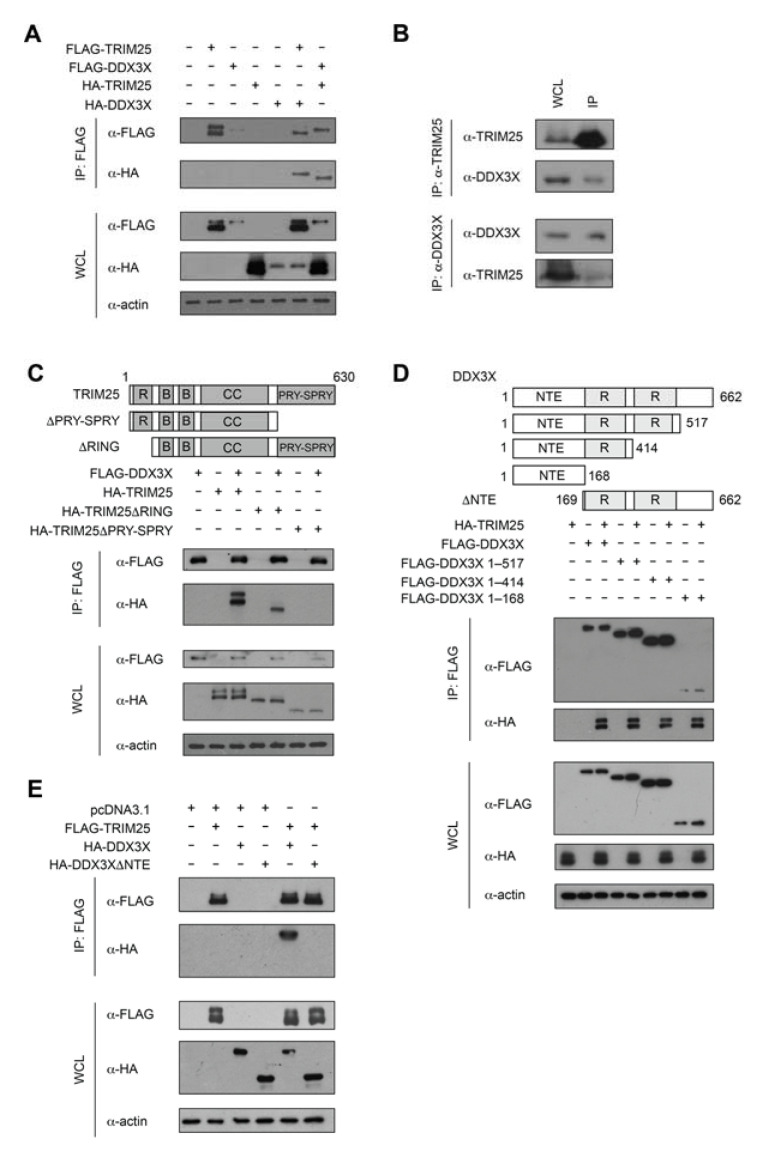
TRIM25 interacts with DDX3X. (**A**) Anti-FLAG immunoprecipitation (IP) of whole-cell lysates (WCL) of HEK293T cells expressing FLAG-TRIM25 and HA-DDX3X, as well as FLAG-DDX3X and HA-TRIM25. Immunoblot (IB) analysis of IP with anti-FLAG and anti-HA antibodies and WCL with anti-FLAG, anti-HA and anti-actin antibodies. (**B**) Interaction between endogenous TRIM25 and endogenous DDX3X in HEK293T cells, with immunoblot analysis of WCL and anti-TRIM25 (top) or anti-DDX3X (bottom) IP. (**C**–**E**) Anti-FLAG IP of WCL of HEK293T cells overexpressing various recombinant proteins. IB analysis of IP with anti-FLAG and anti-HA antibodies and WCL with anti-FLAG, anti-HA and anti-actin antibodies. (**C**) FLAG-DDX3X together with HA-tagged wild-type TRIM25, TRIM25ΔRING or TRIM25ΔPRY-SPRY. In the schematic, R = RING, B = B-box, CC = coiled-coil. (**D**) FLAG-tagged wild-type DDX3X, DDX3X 1–517, DDX3X 1–414 and DDX3X NTE (residues 1–168) constructs together with HA-TRIM25. In the schematic, NTE = N-terminal extension and R = RecA-like domain. (**E**) FLAG-TRIM25 and HA-tagged wild-type DDX3X or DDX3XΔNTE.

To independently validate DDX3X as a binding partner of TRIM25, we performed co-immunoprecipitation of endogenous or overexpressed DDX3X and TRIM25. HEK293T cells overexpressing hemagglutinin (HA)-tagged full-length DDX3X and full-length FLAG-TRIM25 were lysed and immunoprecipitated using anti-FLAG antibody conjugated resin. Western blot analysis of the immunoprecipitates with an anti-HA antibody revealed that HA-DDX3X was immunoprecipitated by FLAG-TRIM25 (Figure 1A). Conversely, in the reciprocal co-immunoprecipitation experiment, HA-TRIM25 was immunoprecipitated by FLAG-DDX3X (Figure 1A). Consistent with the above results, endogenous TRIM25 also co-immunoprecipitated endogenous DDX3X from HEK293T cells (Figure 1B, upper panels), and vice versa (Figure 1B, lower panels), indicating the interaction does occur at physiological expression levels. Together, these results confirm DDX3X is a novel TRIM25 binding partner.

### 2.2. TRIM25 PRY-SPRY Interacts with the DDX3X N-Terminal Extension (NTE)

To map the region of TRIM25 that interacts with DDX3X, we overexpressed FLAG-tagged full-length or truncated DDX3X and HA-tagged full-length or truncated TRIM25 in HEK293T cells (Figure 1C). Cell lysates immunoprecipitated with anti-FLAG resin and subjected to Western blotting using anti-HA antibody revealed that the TRIM25 PRY-SPRY domain (residues 419–630), but not the RING domain (residues 1–84), was required for the interaction with DDX3X (Figure 1C). To map the corresponding region of DDX3X that interacts with TRIM25, we performed a similar experiment with overexpressed HA-tagged full-length TRIM25 and FLAG-tagged full-length DDX3X or DDX3X truncations in HEK293T cells (Figure 1D). The removal of up to 248 residues from the C-terminus of DDX3X (DDX3XΔ415-662) had no evident impact on the interaction with TRIM25 (Figure 1D), and even the most extensive C-terminal truncation (DDX3XΔ169–662), retained the interaction with TRIM25 (Figure 1D), suggesting the DDX3X N-terminal extension (NTE, residues 1–168) mediates TRIM25 binding. This was confirmed by the inability of overexpressed FLAG-TRIM25 to immunoprecipitate overexpressed DDX3X lacking the NTE in HEK293T cells (Figure 1E). Taken together, our results suggest that the TRIM25 PRY-SPRY domain and the DDX3X NTE mediate the TRIM25-DDX3X interaction.

### 2.3. DDX3X Is a Ubiquitination Substrate of the E3 Ligase TRIM25

**Figure 2:**
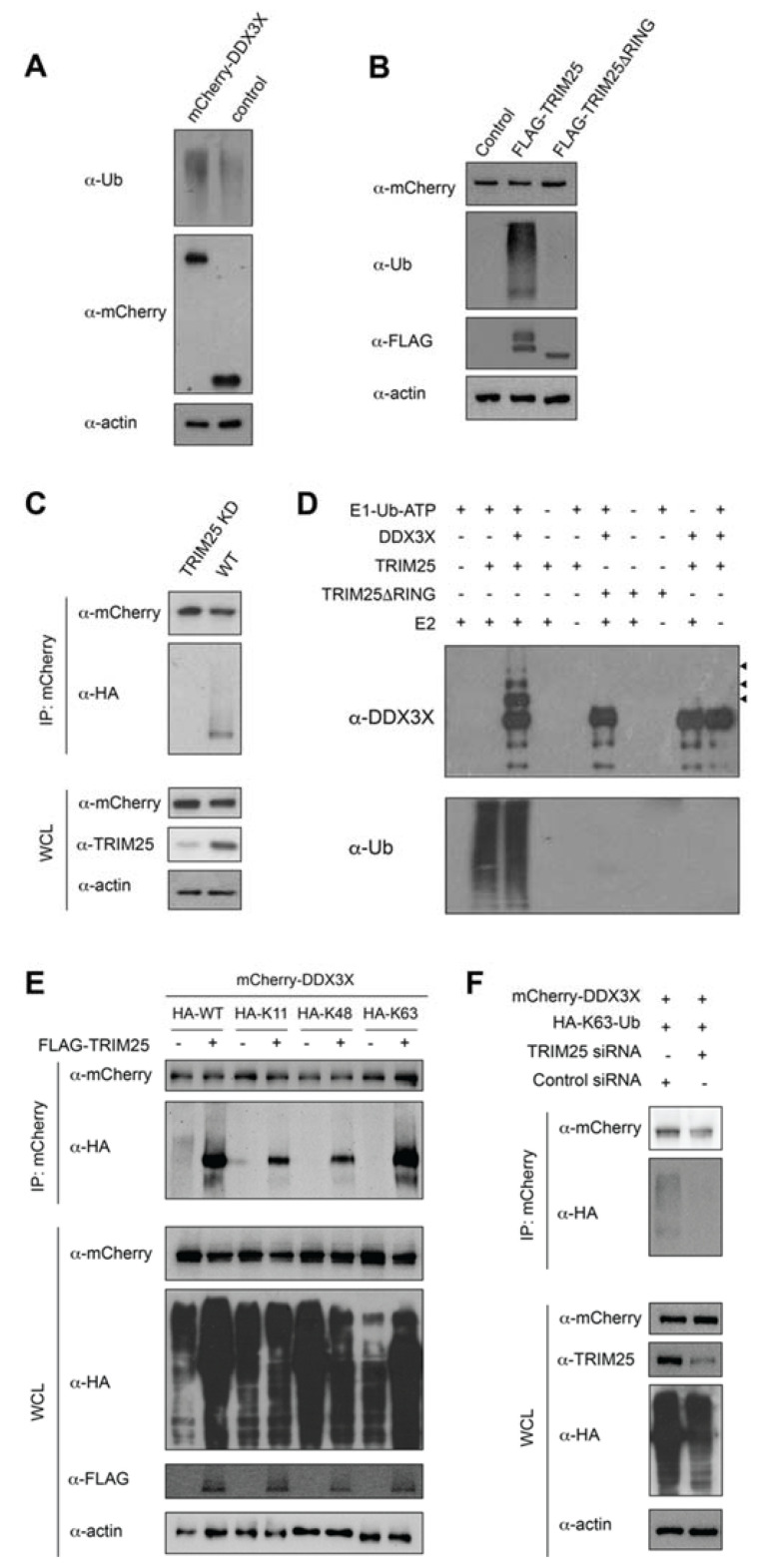
TRIM25 modifies DDX3X with K63-linked polyubiquitin chains. (**A**) Immunoblot analysis of the abundance and ubiquitination (Ub) status of mCherry-DDX3X in HEK293T cells transfected with expression vector for mCherry-DDX3X or empty mCherry vector (control). (**B**) Immunoblot analysis of the abundance (top) and total ubiquitination (middle) of mCherry-DDX3X in HEK293T cells transfected with empty FLAG vector or expression vector for FLAG-TRIM25 or TRIM25ΔRING (bottom). (**C**) Immunoblot analysis of the abundance (first blot) and total ubiquitination (second blot) of mCherry-DDX3X in HEK293T cells transfected to co-express HA-ubiquitin and pSuper-shTRIM25 (TRIM25 KD) or pSuper-shScrambled (WT), assessed after immunoprecipitation with anti-mCherry antibody. (**D**) Immunoblots probed using anti-DDX3X (top) or anti-ubiquitin (bottom) antibody showing in vitro DDX3X ubiquitination reactions consisting of recombinant E1 (Ube1), E2 (UbcH5b), TRIM25, TRIM25ΔRING, ubiquitin (Ub), ATP and wild-type DDX3X(1–580). Arrowheads denote progressively ubiquitinated DDX3X. (**E**) TRIM25 enhanced the K63-linked polyubiquitination of DDX3X. HEK293T cells were transfected with expression plasmids for mCherry-DDX3X and FLAG-TRIM25 or empty FLAG vector, together with HA-tagged wild-type or K11_only_, K48_only_, or K63_only_ ubiquitin mutants. Whole-cell extracts were immunoprecipitated with anti-mCherry antibody-conjugated beads and probed with anti-HA antibody. (**F**) HEK293T cells transfected to express mCherry-DDX3X, HA-tagged K63_only_ ubiquitin and pSuper-shTRIM25 (TRIM25 siRNA) or pSuper-shScrambled (control siRNA) (500 ng). mCherry-tagged proteins were immunoprecipitated with anti-mCherry antibody-conjugated beads probed with anti-HA antibody. The empty FLAG vector (pcDNA3.1) used to subclone was transfected as the “–” for all data. All results are representative of two independent experiments.

Given DDX3X has been identified in proteomic screens as a ubiquitination substrate [23,24,25], together with our new data showing that TRIM25 binds to DDX3X, we tested whether TRIM25 ubiquitinates DDX3X. To test this, we transfected HEK293T cells to express mCherry-DDX3X or mCherry only (control) and immunoblotted using an anti-ubiquitin antibody. Consistent with the literature [24,25], mCherry-DDX3X underwent robust ubiquitination, in contrast to the empty mCherry control (Figure 2A). To test whether this effect was TRIM25-specific, we co-transfected expression constructs encoding overexpressed FLAG-TRIM25 or TRIM25 lacking the catalytic RING domain (TRIM25ΔRING) together with mCherry-DDX3X in HEK293T cells and again probed for ubiquitin. mCherry-DDX3X ubiquitination was markedly enhanced by co-expression of FLAG-TRIM25 compared to FLAG-TRIM25ΔRING or the FLAG-only-containing vector control (Figure 2B), suggesting that TRIM25 is causally involved in the ubiquitination of DDX3X, at least when overexpressed in HEK293T cells.

Next, we knocked down endogenous TRIM25 expression in HEK293T cells by transient transfection with the pSuper RNAi system, prior to co-expression of mCherry-DDX3X and HA-ubiquitin. As a control, we used a scrambled sequence with no greater than 26.3% homology to any known gene in humans. TRIM25 expression was reduced by approximately 90% in cells transfected with pSuper-shTRIM25, compared with those transfected with the pSuper-shScrambled vector control, with no effect on endogenous (actin) or exogenous (mCherry) protein expression (Figure 2C). Consistent with our TRIM25 overexpression data, we observed mCherry-DDX3X ubiquitination in cells expressing endogenous levels of TRIM25, but not in TRIM25-depleted cells (Figure 2C). These results indicate that endogenous TRIM25 ubiquitinates DDX3X in HEK293T cells.

To confirm that DDX3X is a direct substrate of TRIM25, we performed ubiquitination assays using a minimally reconstituted in vitro ubiquitination system. We incubated purified recombinant E1 (Ube1), E2 (UbcH5b) and E3 (TRIM25 or catalytically inactive TRIM25ΔRING) with ubiquitin, DDX3X(1–580) and ATP at 30 °C for 1 h, followed by SDS-PAGE and Western blot analysis. We observed higher molecular weight species consistent with DDX3X polyubiquitination in the presence of full-length TRIM25, but not TRIM25ΔRING (Figure 2D), demonstrating that the catalytic RING domain of TRIM25 is essential for the ubiquitination of DDX3X. Moreover, the ubiquitinated products were not detected when either E1, E2, TRIM25 or DDX3X were omitted from the reaction. Together, these data indicate that DDX3X is a direct ubiquitination substrate of TRIM25.

### 2.4. TRIM25 Mediates K63-Linked Ubiquitination of DDX3X

The fate of a ubiquitinated protein is determined by the ubiquitin chain linkage type that is conjugated to it. TRIM25 has previously been reported to catalyse K48- or K63-linked polyubiquitination of substrates [11,16,26]. To identify the ubiquitin-linkage type that TRIM25 conjugates to DDX3X, we transfected HEK293T cells to express mCherry-DDX3X, FLAG-TRIM25 and either wild-type HA-ubiquitin (HA-WT) or HA-ubiquitin mutants bearing only single lysine (K) residues (K48_only_, K63_only_ or, as a control, K11_only_), followed by immunoprecipitation with anti-mCherry and assessment of ubiquitination by Western blot. We found that TRIM25 was able to form polyubiquitin chains on DDX3X in the presence of K63_only_ ubiquitin but not K11_only_ or K48_only_ ubiquitin, suggesting DDX3X undergoes polyubiquitination and K63 is specifically required for the ubiquitin chain linkage (Figure 2E). This finding was further confirmed in TRIM25 knockdown cells, where HEK293T cells depleted of TRIM25 exhibited greatly reduced K63-linked polyubiquitination of DDX3X compared to cells transfected with the scrambled shRNA expression plasmid (Figure 2F). Our results indicate that TRIM25 specifically conjugates K63-linked polyubiquitination of DDX3X in HEK293T cells.

### 2.5. TRIM25 Ubiquitinates DDX3X Residue K55

**Figure 3:**
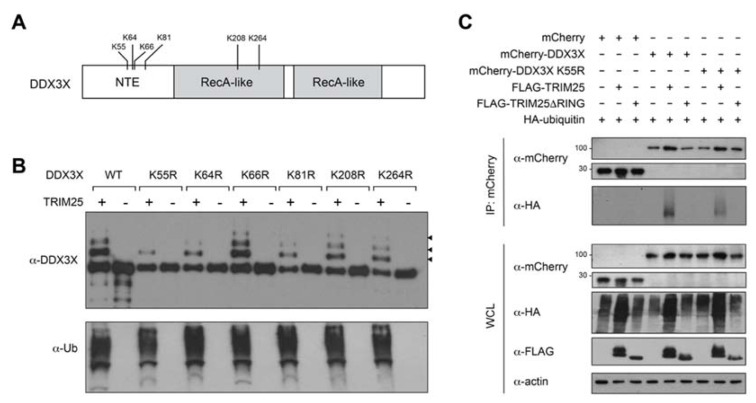
TRIM25 ubiquitinates DDX3X at lysine residue 55. (**A**) DDX3X domain schematic showing location of di-glycine-modified DDX3X residues identified in this study and by others [25]. NTE = N-terminal extension. (**B**) Immunoblot analysis of DDX3X in vitro ubiquitination assay with recombinant E1, E2 (UbcH5b), ubiquitin (Ub) and ATP together with wild-type (WT) or lysine (K) > arginine (R) substituted DDX3X. Arrowheads denote progressively ubiquitinated DDX3X. (**C**) Immunoblot analysis (with anti-mCherry antibody) of the abundance (top) and total ubiquitination (anti-HA antibody, second blot) of mCherry-tagged WT and K55R substituted DDX3X in HEK293T cells transfected with empty FLAG vector or expression vector for FLAG-TRIM25 or TRIM25ΔRING, assessed after immunoprecipitation with anti-mCherry antibody.

To investigate the functional significance of DDX3X’s ubiquitination by TRIM25, we began by determining the TRIM25-mediated ubiquitin conjugation site/s on DDX3X. DDX3X residues K55, K208 and K264 were previously reported as DDX3X ubiquitination sites in resting HEK293T cells [25]. Following in vitro ubiquitination assays and mass spectrometry analysis, we further identified residues K64, K66 and K81 as putative DDX3X ubiquitination sites (Figure 3A; Supplementary Table S2; Supplementary Figure S3A–F. To ascertain whether TRIM25 mediates the ubiquitination of any of these sites, we individually substituted each of the six aforementioned DDX3X lysine residues to arginine, then expressed and purified the recombinant mutant DDX3X proteins together with TRIM25 (Supplementary Figure S1A) and performed additional in vitro ubiquitination assays (Figure 3B). SDS-PAGE analysis of the ubiquitination reactions revealed that in comparison to wild-type DDX3X and DDX3X(K66R), the DDX3X mutant proteins K64R, K81R, K208R and K264R showed modestly reduced TRIM25-mediated ubiquitination, suggesting these may be minor modification sites. Strikingly, however, the K55R substitution caused a near-complete loss of TRIM25-mediated ubiquitination (Figure 3B). This was not due to structural aberrations that might have arisen from the amino acid substitution itself, as we confirmed using circular dichroism that the secondary structure of recombinant DDX3X K55R was essentially identical to wild-type DDX3X (Supplementary Figure S1B). Furthermore, the RNA-dependent ATPase activity of DDX3X(K55R) was indistinguishable from wild-type, in contrast to an established ATPase deficient mutant, DDX3X(K230E) (Supplementary Figure S1C). These data indicate that DDX3X residue K55 is a predominant acceptor site for TRIM25-mediated ubiquitination in vitro.

To determine whether K55 is a bona fide acceptor site for TRIM25-mediated ubiquitination in cells, we co-expressed mCherry-tagged wild-type DDX3X or DDX3X(K55R) in HEK293T cells with both HA-ubiquitin and either FLAG-TRIM25, FLAG-TRIM25ΔRING or empty FLAG-vector (Figure 3C). Cell lysates immunoprecipitated with anti-mCherry antibody and immunoblotted with an anti-ubiquitin antibody confirmed that the DDX3X(K55R) substitution showed reduced TRIM25-mediated ubiquitination compared to wild-type DDX3X (Figure 3C), and in both cases ubiquitination was explicitly TRIM25-RING-dependent. As a control, we confirmed that DDX3X(K55R) immunoprecipitated overexpressed TRIM25 in HEK293T cells similar to wild-type DDX3X (Supplementary Figure S1D), suggesting that the K55R substitution has no effect on the interaction itself. Taken together, these results suggest that TRIM25 ubiquitinates DDX3X residue K55 in a RING-dependent manner.

### 2.6. TRIM25 and DDX3X Amplify RIG-I-Mediated IFNB1 Induction

**Figure 4:**
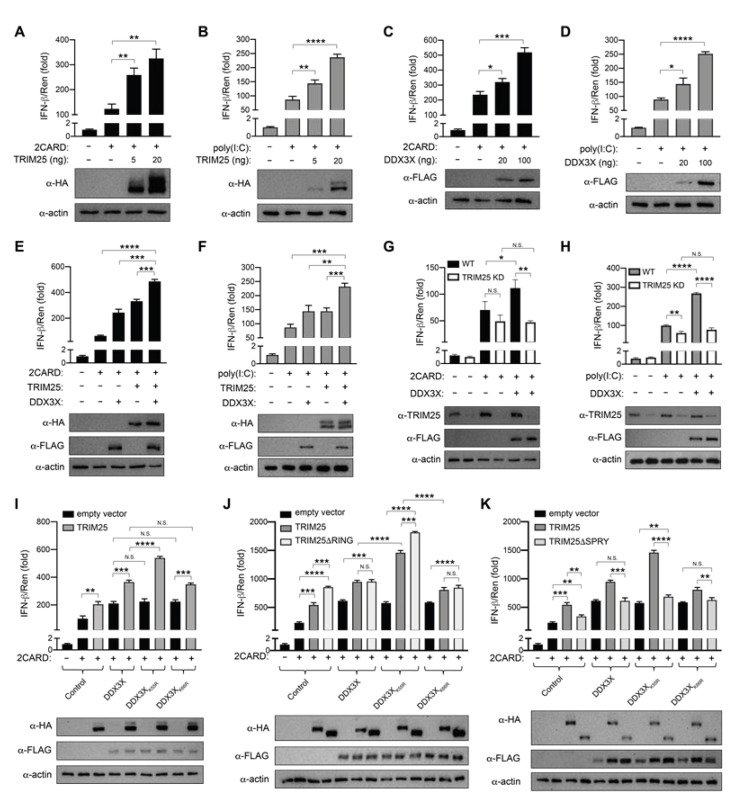
TRIM25 and DDX3X cooperatively enhance *IFNB1* promoter induction. Activity of firefly luciferase expressed under the control of the *IFNB1* promoter (pGL3-IFN-β1) measured after 24 h of RLR signalling cascade activation with RIG-I 2CARD (**A**,**C**,**E**,**G**,**I**,**J**,**K**) or 18 h of poly(I:C) stimulation (**B**,**D**,**F**,**H**) in HEK293T cells transfected to express recombinant proteins as indicated. (**A**,**B**) Increasing amounts of HA-TRIM25 expression vector or control FLAG vector. (**C**,**D**) Increasing amounts of FLAG-DDX3X expression vector or control FLAG vector. (**E**,**F**) FLAG-DDX3X and HA-TRIM25. (**G**,**H**) FLAG-DDX3X and HA-TRIM25 in cells transfected with pSuper-shTRIM25 (TRIM25 KD) or pSuper-shScrambled (WT). (**I**) FLAG-tagged WT, K55R or K66R DDX3X and HA-TRIM25. (**J**) FLAG-tagged WT, K55R or K66R DDX3X and HA-tagged WT or ΔRING TRIM25. (**K**) FLAG-tagged WT, K55R or K66R DDX3X and HA-tagged WT or ΔPRY-SPRY TRIM25. Firefly luciferase results normalised to the activity of Renilla luciferase internal control. The empty vector pcDNA3.1 FLAG was transfected as the “–” for all data. All results are representative of three independent experiments. Representative anti-FLAG, anti-HA and anti-actin immunoblots are shown. Graphs show the mean ± SD of three replicates. Statistical significance was determined using one-way ANOVA with Tukey’s multiple comparisons test and assessed based on the *p* value: NS *p* > 0.05, * *p* ≤ 0.05, ** *p* ≤ 0.01, *** *p* ≤ 0.001, and **** *p* ≤ 0.0001.

Given that both TRIM25 and DDX3X are reported to play individual roles in *IFNB1* gene induction following RIG-I activation [11,16,27,28,29], we tested whether the TRIM25:DDX3X interaction modulates IFN-β production. We first confirmed the individual contribution of both proteins to IFN-β production following stimulation with RIG-I 2CARD (which constitutively activates RIG-I-dependent signalling) [11,14], or poly(I:C) [16,30] (a synthetic mimic of viral double-stranded RNA), using luciferase gene reporter assays in HEK293T cells, which do not express endogenous toll-like receptors (TLRs) [31]. Consistent with their activating roles in the literature [11,16,28,29,32], overexpressing the HA-TRIM25 (Figure 4A,B) or FLAG-DDX3X (Figure 4C,D) proteins described above significantly enhanced *IFNB1* reporter activation in HEK293T cells in response to stimulation with RIG-I 2CARD (Figure 4A,C) or poly(I:C) (Figure 4B,D), and both in a dose-dependent manner. Next, we co-expressed both TRIM25 and DDX3X in HEK293T cells and again monitored *IFNB1* gene induction using a luciferase reporter. Following stimulation from either RIG-I 2CARD (Figure 4E) or poly(I:C) (Figure 4F), TRIM25 and DDX3X co-expression significantly amplified (*p* ≤ 0.001) *IFNB1* gene induction, compared with either TRIM25 or DDX3X alone (Figure 4E,F).

This effect was validated in the same system together with a TRIM25 shRNA expression plasmid or a scrambled shRNA expression plasmid as a control. TRIM25 expression was reduced by ≥50% in cells transfected with the TRIM25 shRNA expression plasmid compared with the scrambled shRNA expression plasmid (Figure 4G,H), the latter of which showed wild-type levels of TRIM25 expression. Following RIG-I 2CARD (Figure 4G) or poly(I:C) (Figure 4H) stimulation, TRIM25 knockdown HEK293T cells showed comparable levels of *IFNB1* gene reporter expression with or without overexpressed DDX3X (Figure 4G,H). However, cells expressing endogenous TRIM25 and overexpressing DDX3X showed a significant increase in *IFNB1* gene induction compared with cells in which DDX3X was not overexpressed (Figure 4G,H). These results confirm that both TRIM25 and DDX3X cooperatively upregulate IFN-β following stimulation by either poly(I:C) or RIG-I 2CARD.

### 2.7. DDX3X K55 Modulates TRIM25:DDX3X Synergistic IFNB1 Induction

Our above overexpression and knockdown experiments reveal the cooperative activation of the *IFNB1* promoter by TRIM25 and DDX3X. We also showed that DDX3X is a substrate of the TRIM25 E3 ligase and is ubiquitinated at K55. To determine whether the DDX3X K55R substitution impacted DDX3X’s ability to synergise with TRIM25, we also tested its effect on *IFNB1* induction. The individual overexpression of DDX3X, DDX3X(K55R) or DDX3X(K66R) (a proximal control which is ubiquitinated by TRIM25 at levels similar to wild-type DDX3X; Figure 3B) led to comparable levels of *IFNB1* gene induction in the presence of RIG-I 2CARD (Figure 4I). However, the addition of TRIM25 further significantly enhanced (*p* ≤ 0.0001) *IFNB1* gene induction when in the presence of DDX3X(K55R) compared to TRIM25:DDX3X or TRIM25:DDX3X(K66R). Taken together, our data suggest that wild-type DDX3X cooperates with TRIM25 to enhance *IFNB1* gene induction, and that DDX3X residue K55 is involved in limiting this effect.

Given the unexpected result that the DDX3X(K55R) mutant, which is no longer ubiquitinated by TRIM25, caused an increase in *IFNB1* gene induction in the presence of TRIM25, we further investigated the association of TRIM25 catalytic activity and DDX3X binding with *IFNB1* gene induction. We transfected HEK293T cells to overexpress DDX3X, DDX3X(K55R) or DDX3X(K66R) with or without wild-type TRIM25, TRIM25 lacking the catalytic domain (TRIM25ΔRING) (Figure 4J) or TRIM25 lacking the substrate binding domain (TRIM25ΔPRY-SPRY) (Figure 4K) and measured *IFNB1* gene induction following stimulation with RIG-I 2CARD. Intriguingly, the overexpression of TRIM25ΔRING alone (Figure 4J) led to a significant (*p* ≤ 0.001) increase in *IFNB1* gene induction compared to wild-type TRIM25 alone, with levels comparable to TRIM25:DDX3X cooperative *IFNB1* gene induction. Also comparable were the levels of *IFNB1* reporter activation by TRIM25ΔRING:DDX3X or TRIM25ΔRING:DDX3X(K66R) (Figure 4J). In contrast, the co-expression of TRIM25 with DDX3X(K55R) caused a further significant (*p* ≤ 0.0001) increase in *IFNB1* gene induction compared to the levels achieved when TRIM25 was co-expressed with either wild-type DDX3X or DDX3X(K66R) (Figure 4J). A further significant (*p* ≤ 0.001) increase in *IFNB1* gene induction was again observed when TRIM25ΔRING was co-expressed with DDX3X(K55R) (Figure 4J). These results suggest that the TRIM25 catalytic RING domain is dispensable for TRIM25-dependent IFN-β stimulation.

Next, we sought to clarify what role, if any, the TRIM25 PRY-SPRY may have in this process. We found that the overexpression of TRIM25ΔPRY-SPRY alone led to a significant decrease in *IFNB1* gene induction compared to wild-type TRIM25 alone, but also when co-expressed with wild-type DDX3X, DDX3X(K55R) or DDX3X(K66R) (Figure 4K). Taken together, these results suggest that cooperative TRIM25:DDX3X *IFNB1* gene induction, which is further enhanced by the DDX3X(K55R) mutant, is dependent on the physical interaction of the two proteins, rather than the catalytic activity of TRIM25.

### 2.8. DDX3X ATPase Activity Modulates DDX3X-TRIM25 Cooperation in IFN-β Signalling

Synergistic activation of the *IFNB1* promoter did not depend on the catalytic activity of TRIM25. However, this left open the possibility that DDX3X’s catalytic activity may modulate this effect. To ascertain whether DDX3X’s catalytic activity is required for TRIM25:DDX3X synergistic *IFNB1* gene induction, we transfected HEK293T cells with TRIM25 and DDX3X or DDX3X variants including DDX3X(K55R) or the ATPase deficient DDX3X mutant (K230E) (Supplementary Figure S1C). We also co-expressed TRIM25 with DDX3X or the DDX3X variants and measured *IFNB1* induction following stimulation with RIG-I 2CARD (Supplementary Figure S2). When TRIM25 was co-expressed with the catalytic dead DDX3X(K230E), cooperative *IFNB1* induction was again significantly (*p* ≤ 0.0001) enhanced. This suggests that DDX3X’s catalytic activity is not required for TRIM25:DDX3X cooperative *IFNB1* induction, but that DDX3X’s ATPase activity may serve as a ‘brake’ to modulate it.

### 2.9. Influenza A Virus NS1 Inhibits TRIM25 Ubiquitination of DDX3X

**Figure 5:**
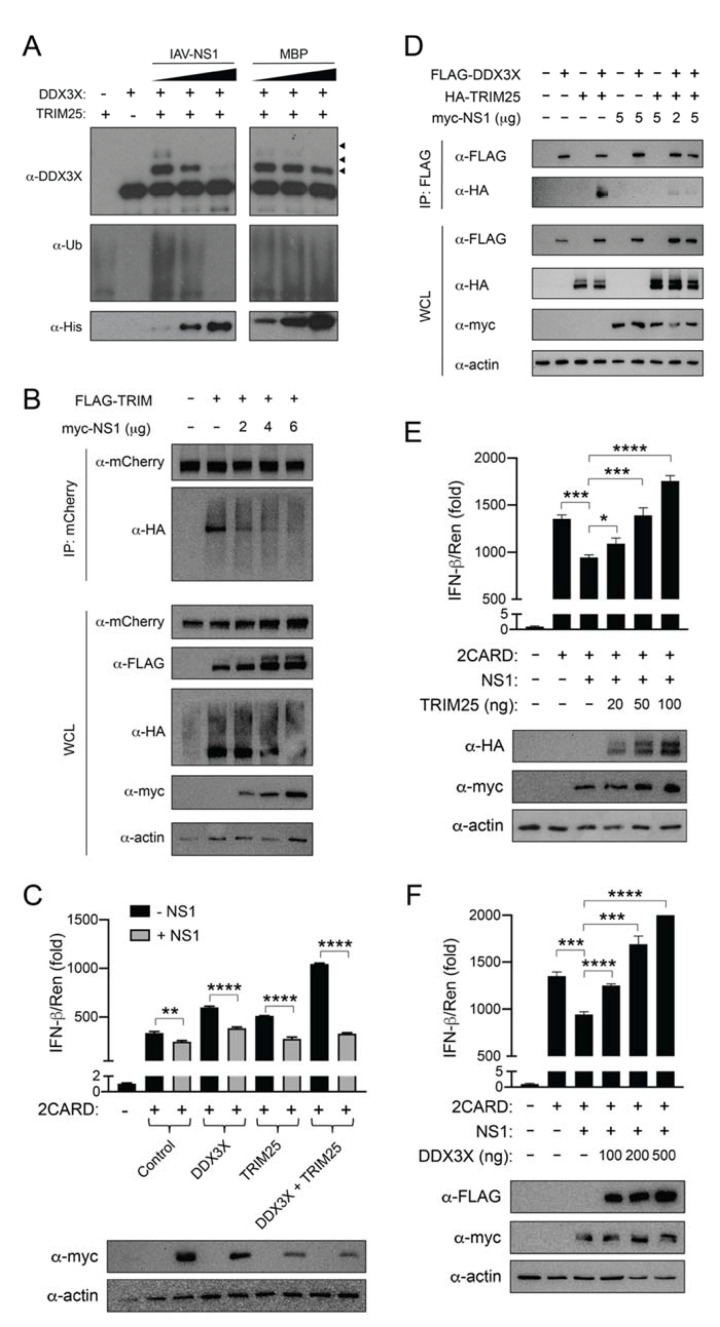
Influenza A NS1 inhibits the TRIM25 catalysed ubiquitination of DDX3X. (**A**) Immunoblot probed using anti-DDX3X antibody showing in vitro DDX3X ubiquitination reactions consisting of recombinant Ube1, UbcH5b, TRIM25, DDX3X(1–580), ubiquitin, ATP and increasing concentrations of influenza A virus non-structural protein 1 (IAV–NS1) (NS1:TRIM25 molar ratio: 1:1, 3:1, 5:1), or, as a specificity control, *E. coli* maltose-binding protein (MBP) (MBP:TRIM25 molar ratio: 1:1, 3:1, 5:1). Arrowheads denote ubiquitinated DDX3X. (**B**) Immunoblot analysis (with anti-HA antibody) of the abundance (first blot) and total ubiquitination (second blot) of mCherry-DDX3X in HEK293T cells transfected with empty vector or expression vector for FLAG-TRIM25, HA-ubiquitin and increasing amounts of myc-NS1 (0–6 μg), assessed after immunoprecipitation with anti-mCherry antibody. (**C**) Activity of firefly luciferase expressed under the control of the *IFNB1* promoter (pGL3-IFN-β1) measured after 24 h of RIG-I 2CARD stimulation in HEK293T cells expressing FLAG-DDX3X and HA-TRIM25 together with control vector (− NS1) or myc-NS1 (+ NS1). (**D**) Anti-FLAG IP of WCL of HEK293T cells overexpressing FLAG-DDX3X together with HA-TRIM25 and increasing amounts of myc-NS1 (0–5 μg). IP analysis with anti-FLAG or anti-HA antibodies and WCL with anti-FLAG, anti-HA and anti-actin antibodies. (**E**,**F**) Firefly luciferase activity expressed from the pGL3-IFN-β1 reporter plasmid after 24 h of RIG-I 2CARD stimulation in HEK293T cells expressing myc-NS1 together with increasing amounts of HA-TRIM25 (0–100 ng) (**E**) or FLAG-DDX3X (0–500 ng) (**F**). Firefly luciferase results normalised to the activity of Renilla luciferase internal control. The empty vector pcDNA3.1 FLAG was transfected as the “–” for all data. All results are representative of three independent experiments. Representative anti-FLAG, anti-HA, anti-myc and anti-actin immunoblots are shown. The graphs show the mean ± SD of three replicates. Statistical significance was determined using one-way ANOVA with Tukey’s multiple comparisons test and assessed based on the *p* value: NS *p* > 0.05, * *p* ≤ 0.05, ** *p* ≤ 0.01, *** *p* ≤ 0.001, **** *p* ≤ 0.0001.

The non-structural protein 1 (NS1) of IAV has previously been shown to bind to TRIM25, thereby preventing its ability to ubiquitinate RIG-I [13,21,33,34]. Therefore, we next investigated the ability of IAV NS1 to inhibit TRIM25-mediated DDX3X ubiquitination. We performed in vitro ubiquitination assays by incubating purified TRIM25 with DDX3X, E1, E2, ATP and increasing amounts of IAV NS1 (NS1:TRIM25 molar ratio of 1:1, 3:1 and 5:1). As expected, recombinant IAV-NS1 fully abrogated TRIM25-mediated ubiquitination of DDX3X in a dose-dependent manner, whereas equivalent increasing concentrations of His_6_-tagged maltose-binding protein (MBP) had no effect on DDX3X ubiquitination by TRIM25 (Figure 5A).

Next, we transfected HEK293T cells to express mCherry-DDX3X, FLAG-TRIM25 and HA-ubiquitin in the presence of increasing amounts of myc-IAV-NS1. Consistent with the in vitro ubiquitination assays, the overexpression of increasing amounts of myc-tagged IAV NS1 suppressed mCherry-DDX3X ubiquitination in a dose-dependent manner (Figure 5B). Collectively, these data suggested that IAV-NS1 can inhibit TRIM25-mediated ubiquitination of DDX3X, consistent with its ability to inhibit TRIM25-mediated ubiquitination of RIG-I [13].

To determine the effect of IAV-NS1 on *IFNB1* induction following RIG-I 2CARD stimulation, we transfected cells to overexpress HA-TRIM25, FLAG-DDX3X or both HA-TRIM25 and FLAG-DDX3X in the presence or absence of myc-tagged IAV-NS1 (NS1:TRIM25 molar ratio of 5:1). IAV-NS1 significantly (*p* ≤ 0.0001) inhibited DDX3X- or TRIM25-augmented IFN-β expression by RIG-I 2CARD and completely abrogated (*p* ≤ 0.0001) the cooperative induction of *IFNB1* upon DDX3X and TRIM25 co-expression (Figure 5C). Western blot analysis of anti-FLAG co-immunoprecipitation products with anti-HA antibody revealed that HA-TRIM25 binding to FLAG-DDX3X (Figure 5D) was reduced in the presence of myc-IAV-NS1. Increasing amounts of TRIM25 (Figure 5E) were able to overcome this effect in a dose-dependent manner. Likewise, in the reciprocal experiment, DDX3X overexpression was also able to overcome the IAV-NS1-mediated inhibition of IFN-β in a dose-dependent manner (Figure 5F). Collectively, these results suggest that the TRIM25-mediated ubiquitination of DDX3X is inhibited by IAV-NS1, and that IAV-NS1 abrogates TRIM25:DDX3X cooperative *IFNB1* induction.

## 3. Discussion

The RLR-mediated innate immune response induces the rapid production of type I and type III IFNs and proinflammatory cytokines in response to RNA virus infection. RLR signalling is modulated by the host to prevent prolonged IFN production, which is linked to the development of autoimmune disease [35,36]. Viruses also modulate RLR signalling to benefit their replication [13,32,37,38,39,40]. Thus, it is imperative we understand the host and viral mechanisms that regulate RLR signalling. Here, we identify a novel interaction between TRIM25 and DDX3X that modulates RIG-I-mediated *IFNB1* gene induction and is inhibited by IAV-NS1. Our new-found association between DDX3X and TRIM25 adds to the growing number of interacting pairs of RNA helicases and TRIM family E3 ligases, supporting an evolutionary connection (e.g., [11,41,42,43]). It is proposed that similar epitopes in the helicase domains of diverse RNA helicases recognise variable loops within the PRY-SPRY domain of TRIM proteins [42]. Intriguingly, however, the DDX3X:TRIM25 interaction does not entirely conform to these conserved rules of engagement. Whilst we show that the TRIM25 PRY-SPRY domain is essential for the interaction, it is the unique unstructured DDX3X NTE, rather than helices within its helicase core that is required for the interaction.

DDX3X contributes to *IFNB1* promoter induction following RIG-I activation through association with MAVS/RIG-I [29], further ‘downstream’ via association with TBK1 and IKKε [27,28,29,44], and at the transcriptional level through direct interaction with the *IFNB1* promoter [28]. The TBK1- and IKKε-mediated IFN-β enhancing functions are independent of DDX3X’s catalytic activity [27,28], but dependent on the DDX3X NTE. DDX3X-enhanced *IFNB1* induction is abrogated by viral proteins such as vaccinia virus K7 [32], which directly binds the DDX3X NTE, and hepatitis B polymerase which sequesters IKKε from binding the DDX3X NTE [45]. Our findings suggest that a TRIM25:DDX3X complex is constitutively present prior to RIG-I activation, and this amplifies *IFNB1* promoter induction in HEK293T cells following immune stimulation with poly(I:C) or RIG-I 2CARD, emulating exposure to invasive viral RNA and activation of the RLR antiviral signalling cascade, respectively. Notably, TRIM25 is an interferon stimulating gene (ISG), whose expression is upregulated during IFN-I signalling [46]. Thus, in addition to its MAVS-, TBK1- and IKKε-IFN-β enhancing roles, we find that in HEK293T cells, DDX3X also enhances *IFNB1* promoter induction during immune stimulation in cooperation with TRIM25. As per DDX3X’s TBK1- and IKKε-IFN-β enhancing roles, this occurs independently of DDX3X’s catalytic activity, which may instead act as a ‘synergistic brake’ under basal conditions.

Our finding that TRIM25 binds to DDX3X adds to the growing number of RLR-regulatory components that TRIM25 interacts with. In response to viral infection, TRIM25 has previously been reported to mediate the K48-linked ubiquitination of MAVS causing its proteasomal degradation to negatively regulate IFN-β production [16], and the K63-linked ubiquitination of the RIG-I receptor [11,14,15]. TRIM25 also binds the zinc finger antiviral protein (ZAP) and upregulates ZAPs antiviral function, but despite delivering K48- and K63-linked ubiquitin to ZAP, ubiquitination and antiviral enhancement were not correlated [47]. Our finding that TRIM25 mediates the K63-linked ubiquitination of DDX3X residue 55, now further extends the number of RLR-regulating substrates that TRIM25 ubiquitinates, and emphasises TRIM25 is a multifunctional E3 ligase. We found that a K55R substitution caused near-complete loss of TRIM25-mediated DDX3X ubiquitination, which unexpectedly enhanced the cooperation between TRIM25 and DDX3X with respect to *IFNB1* induction. This cooperation was further enhanced by TRIM25 lacking the catalytic domain (TRIM25ΔRING), but entirely lost by TRIM25 lacking the substrate binding domain (TRIM25ΔPRY-SPRY), suggesting that the cooperative activation of the *IFNB1* promoter requires a physical interaction between DDX3X and TRIM25. Consistent with this idea, IAV-NS1, which prevented TRIM25 from binding to and ubiquitinating DDX3X, exerted the opposite effect and blocked the cooperative activation of the *IFNB1* promoter. Collectively, these results suggest that the physical interaction between TRIM25 and the DDX3X NTE enhances *IFNB1* induction and that this occurs in a RING-independent manner and is thus independent of TRIM25’s ability to ubiquitinate DDX3X. Our findings underscore the importance of rigorous testing to avoid misinterpreting the link between ubiquitination and a cellular outcome.

Our finding that the ubiquitin acceptor-deficient DDX3X(K55R) further accentuates TRIM25:DDX3X cooperative activation of the *IFNB1* promoter is intriguing. Notably, lysine 55 is within the unique NTE of DDX3X (residues 1–168), an unstructured region rich in post-translational modifications [28,44,48], and bound by multiple host and viral proteins [27,28,32,44,49], many of which influence IFN-β production. Therefore, it is possible that the arginine substitution at DDX3X K55 enhances IFN-β production by modulating one or more key host-protein interaction/s. Alternatively the DDX3X K55R substitution may block an alternate post-translational modification at that site, such as acetylation, which has been observed at K55 in previous studies [48,50] or RNF39-mediated ubiquitination, thereby preventing DDX3X’s proteasomal degradation [51]. Further studies are required to explore these possibilities.

Our finding that IAV-NS1 prevented the TRIM25-mediated ubiquitination of DDX3X was expected given that IAV-NS1 binds to TRIM25 to disable its E3 ligase activity [13]. As per IAV-NS1, the NS1 protein of respiratory syncytial virus (RSV) [18] and the N protein of SARS-CoV [19] also target TRIM25-mediated RIG-I ubiquitination to suppress type I IFN production. Therefore, it is highly likely that these viral proteins also suppress TRIM25-mediated ubiquitination of DDX3X via TRIM25, presumably also to antagonise RLR signalling to promote their replication. However, a transgenic cell line or in vivo model is needed to further clarify this. Understanding the links between TRIM25-mediated ubiquitination of DDX3X and viral replication will have important implications for understanding viral pathogenesis and identifying novel anti-viral therapeutic strategies.

We report that IAV-NS1 blocks the interaction between TRIM25 and DDX3X to prevent their cooperative *IFNB1* induction, revealing a novel mechanism by which IAV-NS1 modulates IFN-β expression following RIG-I activation [13,52,53]. Our study suggests that the IAV-NS1-mediated targeting of TRIM25 serves a dual role in suppressing IFN-β production. It not only prevents the oligomerisation of TRIM25 to disable the TRIM25-mediated ubiquitination of RIG-I [13] but also prevents TRIM25 from binding to DDX3X, both of which serve to suppress IFN-β production. Although it would be ideal to study this IAV immune evasion mechanism in the context of IAV replication, the multiple interacting partners and roles of TRIM25, DDX3X and IAV-NS1 make it difficult to design experiments that will establish direct cause and effect relationships.

Taken together, our results reveal a previously unrecognised connection between TRIM25 and DDX3X that, independent of TRIM25’s RING-dependent ubiquitination, bolsters IFN-β production. These findings add to the growing number of known interacting partners both TRIM25 and DDX3X have within the RLR signalling cascade and emphasises their roles in finely tuning the IFN-β response are broader and much more complex than previously appreciated. We also reveal a new mechanism by which IAV-NS1 antagonises *IFNB1* induction following RIG-I activation. These findings emphasise the elaborate interplay between host and viral proteins that regulate RLR signalling and will have important implications for further research and the interpretation of subsequent findings.

## 4. Materials and Methods

### 4.1. Co-Immunoprecipitation-Coupled Mass Spectrometry

HEK293T cells were transfected to express FLAG-TRIM25 or mock transfected, then harvested 24 h post-transfection using NP40 lysis buffer containing 25 mM Tris [pH 7.5], 150 mM NaCl, 1 mM EDTA and 0.6% (*v/v*) Nonidet™ P-40 supplemented with 10 mM NaF, 1 mM phenylmethylsulfonyl fluoride (PMSF), 2 mM N-ethylmaleimide (NEM) and 1 mM Na_3_VO_4_, then sonicated, clarified by centrifugation, and incubated with anti-FLAG M2 agarose beads (Sigma-Aldrich, St. Louis, MI, USA) for 4 h at 4 °C. The beads were extensively washed with tris-buffered saline at pH 8.0 and boiled in reducing SDS-PAGE sample buffer for 5 min. Supernatants were separated by SDS-PAGE and Coomassie stained, then TRIM25 protein was excised, reduced with 10 mM DTT, alkylated with 40 mM iodoacetamide (at 25 °C) and trypsinised with modified trypsin (Promega, Madison, WI, USA) at a 1:100 enzyme-to-substrate ratio) at 37 °C for 12 h. The resulting tryptic peptides were resolved by reverse-phase liquid chromatography (LC) on 0.075 × 200 mm fused silica capillaries (J&W) packed with ReproSil reverse-phase material (Dr. Maisch GmbH, Ammerbuch, Germany). The peptides were eluted with linear 65 min gradients of 5 to 45% and 15 min at 95% (*v/v*) acetonitrile with 0.1% (*v/v*) formic acid in water at flow rates of 0.25 μL/min. Mass spectrometry (MS) was performed using a hybrid ion-trap mass spectrometer (Orbitrap XL, Thermo Scientific, Waltham, MA, USA) in a positive mode using repetitively full MS scan followed by collision-induced dissociation (CID) of the 7 most dominant ions selected from the first MS scan. Mass spectrometry data were analysed using the Trans-Proteomic Pipeline (TPP) Version 4.4.1 [54]. TPP-processed centroid fragment peak lists in mzXML format were searched against IPI human database version 3.75 (release date 8/2010). The 89,486 proteins were supplemented with their corresponding decoy sequences (as described in http://www.matrixscience.com/help/decoy_help.html). The database searches were performed using X! Tandem with k-score plugin through the TPP. Search parameters include: trypsin cleavage specificity with two missed cleavages, cysteine carbamidomethyl as a fixed modification, methionine oxidation, peptide tolerance and MS/MS tolerance were set at 10 ppm and 0.8 Da, respectively. X! Tandem refinement included semi style cleavage. Peptide and protein lists were generated following Peptide Prophet and Protein Prophet analysis using a protein false discovery rate (FDR) threshold of <1%. Protein IDs were converted to UniProt accessions.

### 4.2. DDX3X Ubiquitination Sites Mass Spectrometry Analysis

TRIM25-mediated DDX3X in vitro ubiquitination mixtures were analysed by SDS-PAGE. Following Coomassie staining, protein bands were excised and subject to in-gel trypsin digestion and LC–MS/MS analysis as described above. MS data were analysed by using MSFragger version 3.1.1 [55] via FragPipe version 14.0 (https://fragpipe.nesvilab.org/). The searches were conducted using MSFragger’s “closed search” configuration against human protein sequences downloaded from UniProt (version: 10/2020). Precursor mass tolerance was set to 50 ppm, fragment mass tolerance was set to 0.8 Da with specific tryptic digestion, peptide length was set to be between 7 to 35 amino acids. Oxidation of methionine, protein N-terminal acetylation and lysine ubiquitination were set as variable modifications. Peptide validation for each modification (1% FDR) was performed following PeptideProphet [56] and iProphet [57] analyses. The modifications and their localisation were validated and using PTMProphet [58]. Only DDX3X ubiquitinated peptides with a localisation probability higher than 0.9 were considered. MS/MS spectra were generated using PDV (version 1.73) [59].

### 4.3. Molecular Cloning

Full-length human DDX3X (residues 1–662) constructs in pmCherry-C1 (EcoRI/KpnI), pcDNA3.1-HA (KpnI/EcoRI) or pcDNA3.1-FLAG (KpnI/EcoRI) for mammalian expression, or DDX3X (residues 1–580) constructs in pCOLD-IV (NdeI/KpnI) with sequence specifying an N-terminal His_6_ tag for bacterial expression have been described previously [49]. Constructs for mammalian expression of full-length human DDX3X, TRIM25 and ubiquitin, or fragments or mutants thereof, fused with N-terminal FLAG, HA or myc-BirA* epitope tags were generated using the pcDNA3.1 vector (Thermo Fisher Scientific, Waltham, MA, USA). The pLIC vector was used for bacterial expression of TRIM25 harbouring an N-terminal His_6_-MBP fusion tag. NS1 from influenza A/PR8/34 was synthesised (GenScript, Piscataway, NJ, USA) and subcloned into the pCOLD bacterial expression vector (Takara, San Jose, CA, USA) with an N-terminal His_6_ fusion tag or the pcDNA3.1 vector (Thermo Fisher Scientific, Waltham, MA, USA) with a myc tag. UbE1 in pET24b with a C-terminal His_6_ tag was a kind gift from Catherine Day (University of Otago). UbcH5b in pET32b with an N-terminal His_6_ tag was a kind gift from Sharad Kumar (University of South Australia). Site-directed mutagenesis was performed using the QuikChange method (Agilent Technologies, Santa Clara, CA, USA). TRIM25-specific small hairpin RNA (shRNA) was produced as described previously [60]. The target was TRIM25 cDNA region 711–729 (5′-GGTGGAGCAGCTACAACAA-3′) and the synthesised oligonucleotide was: 5′-agatctccGGTGGAGCAGCTACAACAAttcaagagaTTGTTGTAGCTGCTCCACCtttttaagctt-3′. A scrambled sequence with less than 26.3% homology to any other human gene was used as a specificity control. This sequence was: 5′-GGGCCGTGGACAACTAAAA-3′, and the synthesised oligonucleotide was: 5′-agatctccGGGCCGTGGACAACTAAAAttcaagagaTTTTAGTTGTCCACGGCCCtttttaagctt-3′. Both shRNA oligonucleotides were flanked with a 5′-BglII and 3′-HindIII restriction site for engineering into mammalian expression vector pSuper.Retro.Puro (OligoEngine, Seattle, WA, USA) to generate the TRIM25-shRNA expression vector, pSuper-shTRIM25, or the scrambled-shRNA expression vector, pSuper-shScrambled. Expression constructs were synthesised by Bioneer Pacific (Daejeon, Korea), and verified by DNA sequencing.

### 4.4. Cell Culture and Transfection

HEK293T cells were cultured in Dulbecco’s Modified Eagle Medium (DMEM) (Gibco, Amarillo, USA) supplemented with 10% (*v/v*) foetal bovine serum (Assay Matrix, Melbourne, Australia), 2 mM L-glutamine and 100 U mL^−1^ penicillin/100 µg mL^−1^ streptomycin (Gibco, Amarillo, TX, USA). Transfections were performed with FuGENE HD (Promega, Madison, WI, USA). For TRIM25 knockdown experiments, HEK293T cells were transfected with 1 µg mL^−1^ pSuper-shTRIM25 or control pSuper-shScrambled using Fugene HD (Promega, Madison, WI, USA). For experiments assessing DDX3X ubiquitination, cells were treated with 25 μM MG132 for 4 h prior to harvesting.

### 4.5. Immunoprecipitation

For immunoprecipitation, cells were thoroughly washed with PBS and lysed in total cell lysis buffer (20 mM Tris-Cl pH 7.4, 1% (*v/v*) IPEGAL^®^ CA-630, 150 mM NaCl), supplemented with 100 µM PMSF, 1 mM Na_3_VO_4_, 50 mM NaF, 2 mM N-ethylmaleimide (NEM), and cOmplete™ EDTA-free protease inhibitor cocktail tablets (Roche, Basel, Switzerland). Lysates were clarified by centrifugation at 16,100 rcf (13,200 rpm) for 15 min at 4 °C, then added to anti-FLAG M2 magnetic beads (Sigma-Aldrich, St. Louis, MI, USA) or anti-mCherry antibody (Abcam, Cambridge, UK #ab167453), anti-TRIM25 (Abcam, Cambridge, UK #ab86365) or anti-DDX3X (Biorbyt, Cambridge, UK #ORB167469) immobilised on Dynabead^®^ Protein G magnetic resin (Life Technologies, Carlsbad, CA, USA). Immunoprecipitation proceeded overnight at 4 °C. Captured proteins were extensively washed with total cell lysis buffer then with PBS and eluted by boiling in 2× Laemmli sample buffer.

### 4.6. Immunoblotting

Proteins resolved by SDS-PAGE were transferred to Immobilon-P PVDF membrane (Millipore, Burlington, VT, USA). Blots were probed with anti-DDX3X (GeneTex #GTX110614; 1:1000 *v/v*), anti-TRIM25 (Abcam #ab86365; 1:1000 *v/v*), anti-FLAG (Cell Signalling Technology, Danvers, USA, CST #8146S, #73916S; 1:1000 *v/v*, SCBT #sc-166355; 1/200 *v/v*), anti-HA (CST #2367S, #3724S; 1:1000 *v/v*), anti-mCherry (1:1000 *v/v*), anti-myc (CST #5605S; 1:1000 *v/v*), anti-ubiquitin (CST #3936; 1:1000 *v/v*) or anti-β-actin HRP-conjugate (CST #5125; 1:5000 *v/v*) antibodies, followed by HRP-conjugated anti-rabbit (Millipore, Burlington, USA #12-348; 1:10,000 *v/v*) or anti-mouse (CST #7076; 1:10,000 *v/v*) antibodies. The 5% (*w*/*v*) skim milk in TBS with 0.1% (*v/v*) Tween-20 was used to block and wash membranes and was used as the antibody diluent.

### 4.7. Luciferase Gene Reporter Assays

HEK293T cells were transfected to co-express the pGL3 reporter construct encoding firefly luciferase under control of the *IFNB1* promoter together with the constitutive pTK-renilla luciferase construct as a normalisation control. Expression constructs were co-transfected with the reporter constructs. The stimulant was transfected either concurrently (pEF-BOS-RIG-I 2CARDs, 100 ng per well of a 24-well plate or scaled accordingly), or 6 h after reporter transfection (LMW poly(I:C); Invivogen, San Diego, CA, USA, #tlrl-picw, 1 μg added per well of a 24-well plate or scaled accordingly) and incubated overnight. Cells were harvested 24 h after reporter transfection, resulting in 24 h of RIG-I 2CARD stimulation or 18 hrs of poly(I:C) stimulation. Luciferase activity was measured using the Dual-Luciferase^®^ Gene Reporter Assay System (Promega, Madison, WI, USA) and a Clariostar plate reader (BMG LabTech, Ortenberg, Germany) equipped with a luminescence detector and liquid injection system. Measurements were performed in triplicate and are representative of at least three independent biological replicates. Summary statistics and one-way ANOVA with Tukey’s multiple comparisons test for significance (*p* ≤ 0.05) were calculated using GraphPad Prism^®^ 8 software (San Diego, CA, USA.)

### 4.8. Recombinant Protein Expression and Purification

His_6_-DDX3X(1–580) and His_6_-DDX3X(1–580) lysine mutants were expressed and purified as described previously [49]. His_6_-UbE1 was expressed and purified as described previously [61]. His_6_-UbcH5b, His_6_-MBP-TRIM25 and His_6_-IAV-NS1 were expressed at 16 °C in *E. coli* strain BL21(DE3) following induction at OD_600nm_ = 0.6 with 1 mM isopropyl 1-thio-β-d-galactopyranoside (IPTG). 20 h post-induction, bacteria were harvested by centrifugation and resuspended in lysis buffer (20 mM Tris-Cl pH 8.0, 500 mM NaCl, 10% (*v/v*) glycerol, 10 mM imidazole, 0.5 mM TCEP). Proteins were extracted by sonication and applied to Ni-NTA Superflow resin (Qiagen, Hilden, Germany). Resin was extensively washed and bound proteins were eluted using elution buffer (lysis buffer + 300 mM imidazole). For His_6_-NS1, resin was extensively washed with Buffer B (20 mM Tris-Cl pH 7.4, 3 M NaCl) to remove bound RNA, and washes were monitored until A_260nm_ reached zero. Eluate was applied to a Superdex 200 16/60 size exclusion chromatography (SEC) column pre-equilibrated in SEC buffer (20 mM Tris-Cl pH 7.4, 150 mM NaCl, 10% glycerol, 1 mM TCEP). His_6_-MBP-TRIM25 was incubated with TEV protease to cleave the N-terminal His_6_-MBP fusion tag, then applied once more to a Superdex 200 16/60 size exclusion chromatography column pre-equilibrated in SEC buffer. Purified His_6_-DDX3X(1–580) and DDX3X(1–580)K55R were structurally and functionally verified as previously described [49]. Recombinant TRIM25, Ube1 and UbcH5b were validated enzymatically, and all proteins were verified by SDS-PAGE and immunoblot.

### 4.9. Circular Dichroism

All measurements were performed in 20 mM Tris, 150 mM NaCl, 0.75 mM TCEP, pH 8.0 using a thermostatically controlled 0.1 cm pathlength cuvette at 20 °C and a Jasco J-815 spectrometer. Scans were taken between 190 and 250 nm at a scan rate of 0.5 nm s^−1^ with 5 accumulations. Sample concentrations were 0.1–0.2 mg/mL. Mean ellipticity values per residue (θ) were calculated as θ = (3300 × m × ΔA)/(lcn), where l is the path length (0.1 cm), n is the number of residues, m is the molecular mass in Daltons, and c is the protein concentration in mg/mL.

### 4.10. ATPase Assays

DDX3X-mediated ATP hydrolysis was measured as described previously [62]. Briefly, reactions consisted of 30 nM DDX3X or mutants, ATPase reaction buffer (20 mM Tris-Cl pH 7.5, 1.5 mM DTT, 1.5 mM MgCl_2_), 0.02 mg/mL poly(I:C), and 0.3 mM ATP (Sigma-Aldrich, St. Louis, MI, USA). After 60 min at room temperature, 100 μL Biomol^®^ Green (Enzo Life Sciences, Farmingdale, NY, USA) was added. Following a 15 min incubation at room temperature, absorbance (620 nm) was measured using a Clariostar plate reader (BMG Labtech, Ortenberg, Germany). A standard curve, generated using serially diluted (0–40 μM) phosphate standards (Enzo Life Sciences, Farmingdale, NY, USA), enabled the quantitation of free inorganic phosphate (Pi) generated by DDX3X ATPase activity.

### 4.11. In Vitro Ubiquitination Assays

Recombinant His_6_-DDX3X (0.75 μM), TRIM25 (with His_6_-MBP-tag cleaved, 0.75 μM), Ube1-His_6_ (0.25 μM), His_6_-UbcH5b (0.75 μM) and His_6_-ubiquitin (5 μM, Boston Biochem, Cambridge, UK,) were incubated in a final volume of 20 μL in 1× ubiquitination reaction buffer (30 mM HEPES pH 7.4, 5 mM MgCl_2_·6H_2_O, 0.2 mM DTT) using a Thermomixer Comfort fitted with a 0.6 mL tube heating block (Eppendorf, Hamburg, Germany) set to 30 °C and 650 rpm for 1 h. Reactions were stopped by boiling with 2× Laemmli sample buffer prior to SDS-PAGE and immunoblotting. For inhibition experiments, His_6_-IAV-NS1 or His_6_-MBP (0.75–3.75 μM) were included in the reaction.

## Data Availability

The mass spectrometry proteomics data have been deposited to the ProteomeXchange Consortium via the PRIDE partner repository with the dataset identifier PXD028122.

## Supplementary Materials

The following are available online at https://www.mdpi.com/article/10.3390/ijms22169094/s1.
