# Supplementary material for: TRIM25 and DEAD-Box RNA Helicase DDX3X Cooperate to Regulate RIG-I-Mediated Antiviral Immunity
Source: Int J Mol Sci. 2021 Aug 23;22(16):9094. doi: 10.3390/ijms22169094 (PMC8396550; doi:10.3390/ijms22169094)
Supplement: Supplementary file 1 [file ijms-22-09094-s001.zip › ijms-1337450-Supplemental.pdf]

Supplementary Information

TRIM25 and DEAD-box RNA helicase DDX3X cooperate to regulate RIG-I–mediated antiviral immunity

Authors: Sarah C. Atkinson, Steven M. Heaton, Michelle D. Audsley, Oded Kleifeld, Natalie A. Borg

Content

Supplementary Figure S1-S3

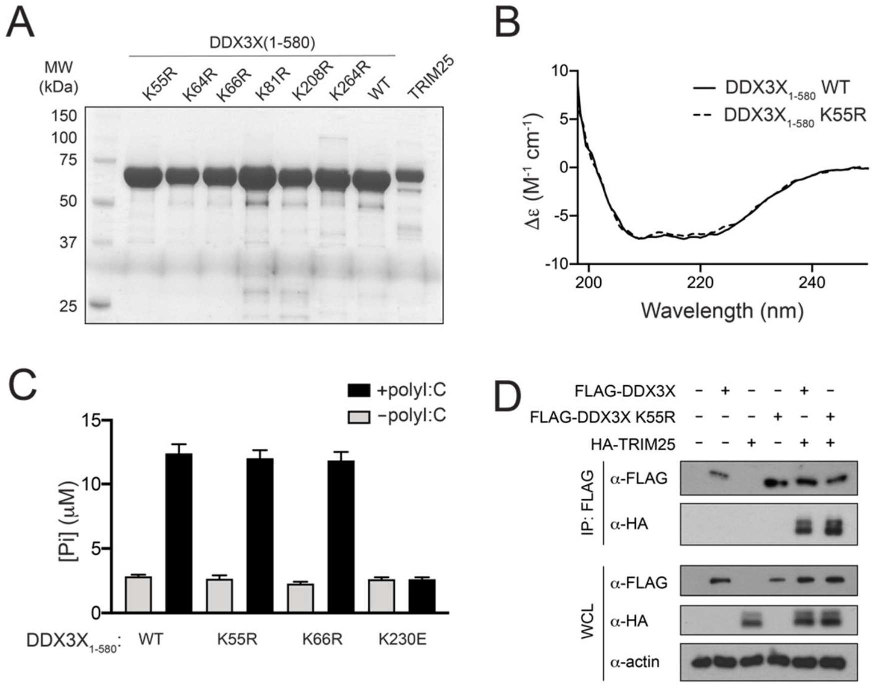

Supplementary Figure S1. Integrity analysis of recombinant DDX3X mutant proteins. **(A)** SDS-PAGE analysis of recombinant WT TRIM25, DDX3X and DDX3X mutant proteins. **(B)** Circular dichroism analysis of the secondary structure of recombinant DDX3X K55R compared to wild-type DDX3X. **(C)** ATPase activity, in both the presence/absence of Poly(I:C), of WT DDX3X, DDX3X(K55R), DDX3X(K66R) and the ATPase deficient mutant DDX3X(K230E) (control). **(D)** α-FLAG immunoprecipitation (IP) of whole cell lysates (WCL) of HEK293T cells expressing HA-TRIM25 and FLAG-DDX3X or FLAG-DDX3X(K55R). Unprocessed original scans of blots can be found in Supplementary Figure 34.

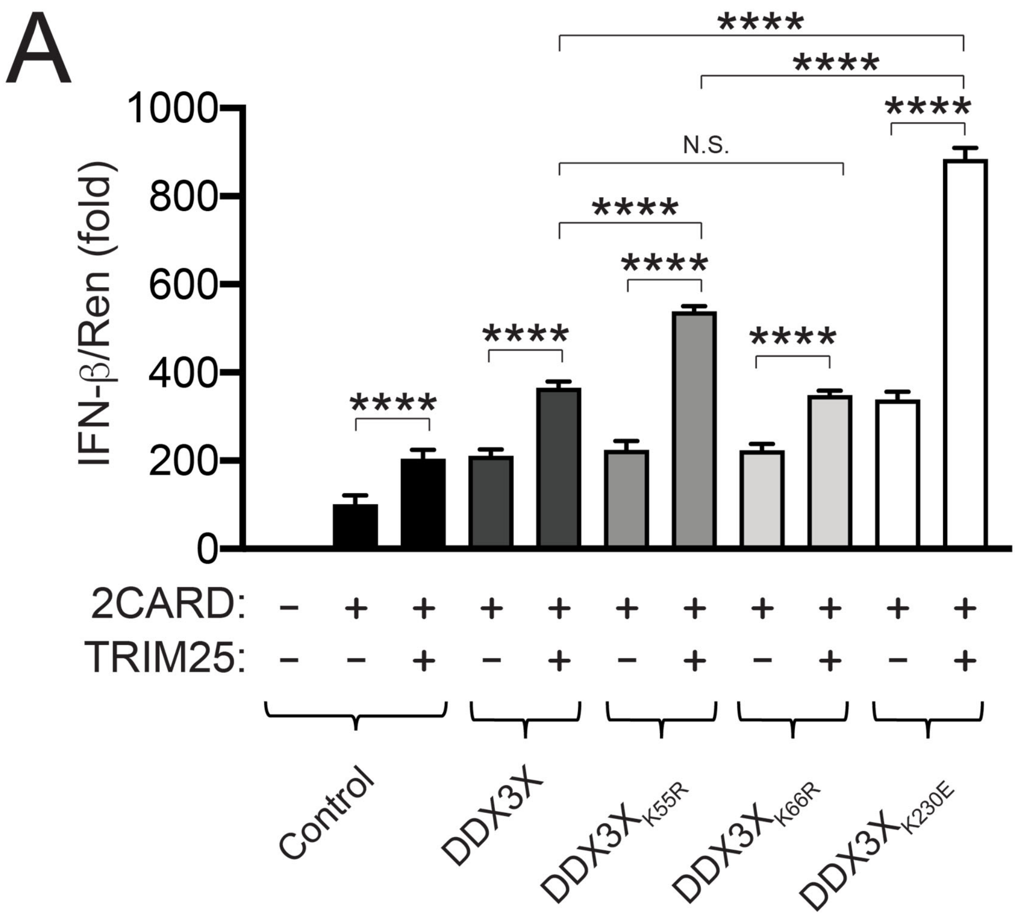

Supplementary Figure S2. TRIM25 and catalytic dead DDX3X(K230E) cooperatively enhance *IFNB1* promoter induction. **(A)** Activity of firefly luciferase expressed under the control of the *INFB1* promoter (pGL3-IFN-β1) measured after 24 h of RLR signalling cascade activation with. RIG-I 2CARD in HEK293T cells transfected to express HA-TRIM25 and FLAG-tagged WT, K55R, K66R or K230E DDX3X. Results are representative of three independent experiments. Statistical significance was determined using one-way ANOVA with Tukey's multiple comparisons test and assessed based on the P value: NS $P > 0.05$, ****$P \leq 0.0001$.

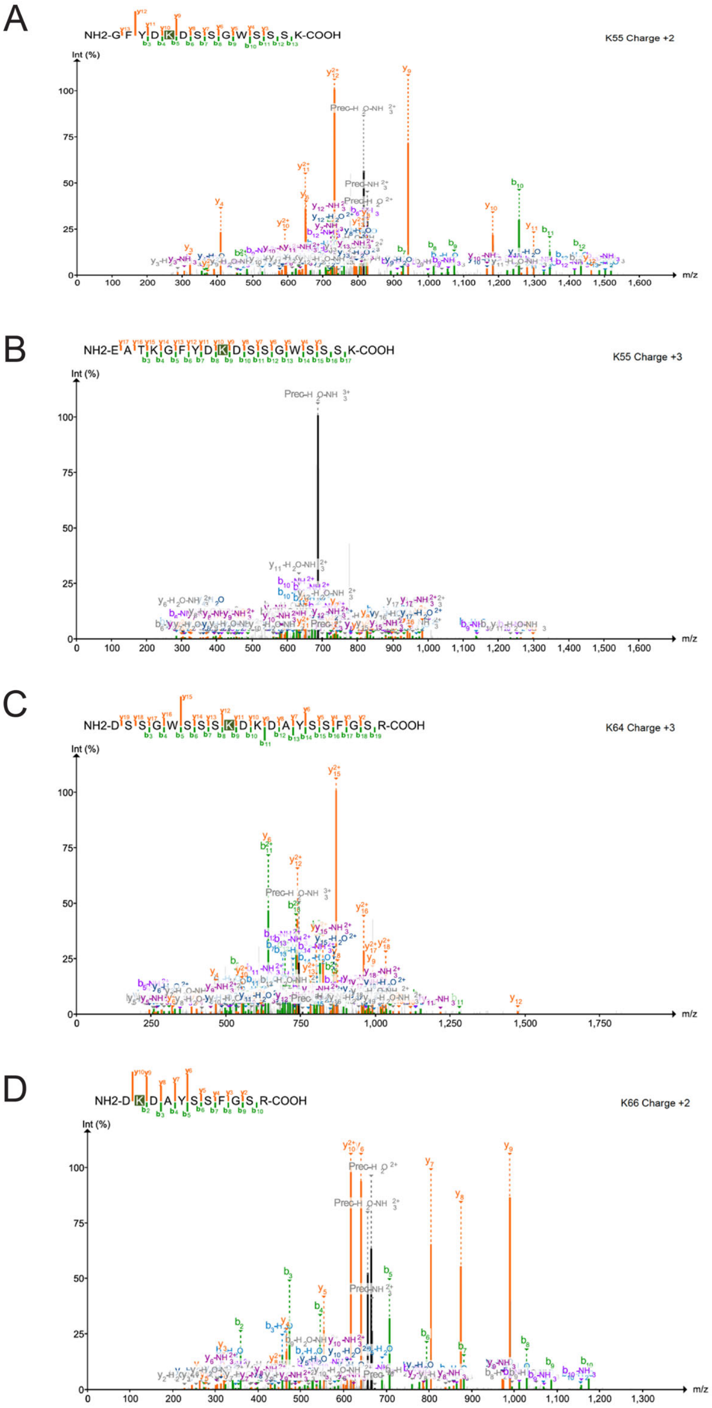
A
NH2-GFYDKDSSGWSSSK-COOH
K55 Charge +2
Int (%)
m/z
B
NH2-EATKGFYDKDSSGWSSSK-COOH
K55 Charge +3
Int (%)
m/z
C
NH2-DSSGWSSSKDKDAYSSFGSR-COOH
K64 Charge +3
Int (%)
m/z
D
NH2-DKDAYSSFGSR-COOH
K66 Charge +2
Int (%)
m/z

E

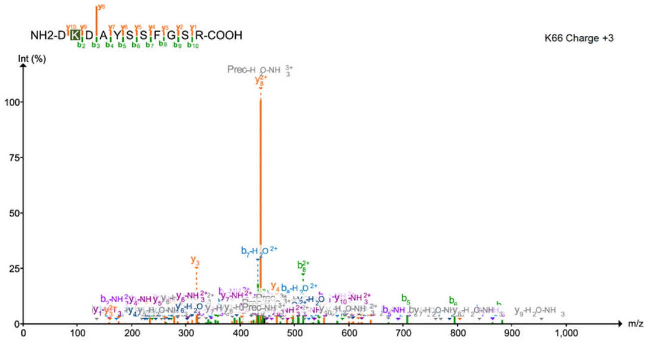

F

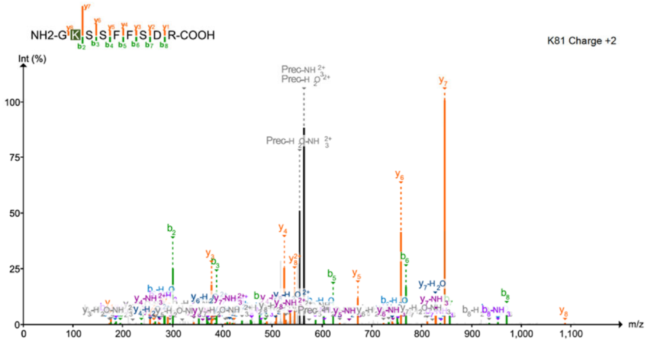

Supplementary Figure S3. Tandem mass spectrometry (MS/MS) spectra obtained from DDX3X ubiquitinated peptides. Spectra annotation was done using PDV package (https://doi.org/10.1093/bioinformatics/bty770). The modified lysine in each spectrum is highlighted with a dark green background. Matched ions are coloured in orange for y-ions, and in green for b-ions. The spectra revealing putative DDX3X ubiquitination sites at lysine (K) **(A,B)** 55, **(C)** K64, **(D,E)** K66 and **F)** K81.
